# Occupational physical activity and cardiovascular disease mortality in the United States, 1988–2019

**DOI:** 10.1186/s12889-024-21225-x

**Published:** 2025-01-07

**Authors:** Tong Xia, Liwei Chen, Jian Li

**Affiliations:** 1 Department of Epidemiology, Fielding School of Public Health, University of California Los Angeles, Los Angeles, CA 90095 USA; 2 Department of Environmental Health Sciences, Fielding School of Public Health, University of California Los Angeles, Los Angeles, CA 90095 USA; 3 School of Nursing, University of California Los Angeles, Los Angeles, CA 90095 USA; 4 Department of Public health Nursing, Faculty of Public health, Mahidol University, Bangkok, 10400 Thailand

**Keywords:** Cardiovascular disease, Cohort study, Mortality, Occupational physical activity

## Abstract

**Background:**

Although leisure time physical activity (LTPA) is a beneficial factor for cardiovascular disease (CVD) mortality, relationships between occupational physical activity (OPA) and CVD mortality are inconclusive. We aimed to examine prospective associations of OPA with CVD mortality using a large representative sample of adult workers in the United States (US), and explore how socioeconomic status (SES) may influence these associations.

**Methods:**

This cohort study included US workers (≥ 18 years) participating in the 1988 National Health Interview Survey (NHIS) and passively followed until December 31, 2019. Time (minutes/week) on strenuous OPA (e.g., lifting, pushing, or pulling heavy objects) was assessed at baseline by a questionnaire and categorized into 4 groups [i.e., none, low, medium, and high]. CVD mortality was identified by International Classification of Diseases, Tenth Version (ICD–10) and collected by the National Death Index database. We examined the association of OPA with CVD mortality using multivariable Cox proportional hazard regressions, controlling for age, sex, race/ethnicity, marital status, education, annual household income, occupation type, and pre-existing cardiometabolic disorders.

**Results:**

In 28,604 participants (46.2% women; mean age 37.86 years), adjusted hazard ratios (95% CIs) of none, low, medium, and high OPA groups were 1.39 (1.01–1.91), 1.00 (reference), 1.18 (0.83–1.66) and 1.58 (1.12–2.22) for CVD mortality. The associations were stronger in workers with low education level (i.e., high school or less) [estimates of none, low, medium, and high OPA groups were 1.74 (1.09–2.78, *P* = 0.02), 1.00, 1.49 (0.92–2.42), and 1.87 (1.16–3.00)] or annual household income <$30,000 [estimates of OPA groups were 1.73 (1.16–2.56), 1.00, 1.29 (0.83–2.01), and 1.73 (1.14–2.65)].

**Conclusions:**

We observed that workers with either high or no strenuous OPA had higher CVD mortality compared to those with low strenuous OPA, demonstrating a U-shaped association in the US. This association was particularly pronounced among workers with lower SES.

**Supplementary Information:**

The online version contains supplementary material available at 10.1186/s12889-024-21225-x.

## Background

Cardiovascular diseases (CVDs) are highly prevalent and are the leading cause of death in the United States (US) [1]. From 2017 to 2020, 48.6% (127.9 million) of adults in the US had CVD [1]. In addition, the estimated direct and indirect costs for CVD were about $407.3 billion from 2018 to 2019, representing approximately 13% of total US healthcare expenditures [1]. Cumulative evidence has shown that higher leisure time physical activity (LTPA) could decrease CVD risk and mortality [2–4]. However, recent evidence suggested the effects of occupational physical activity (OPA) on CVD may be different from LTPA [3, 5, 6]. More importantly, results on the association between OPA and CVD mortality were contradictive, with studies showing the positive [7, 8], inverse [9, 10], and null [11, 12] associations.

Up to now, only one study investigated associations of OPA with CVD mortality in the US [13]. Using data from National Institutes of Health–American Association of Retired Persons (AARP) Diet and Health Study among 322,126 retired persons aged ≥ 50 years in the US, a J-shaped associations between years of heavy labor and CVD mortality was observed [13]. However, one important feature of this study was that only retirees were eligible for participation, excluding those who were currently employed [13]. Given inconsistent findings from the previous studies and limited studies in the US regarding the association of OPA with CVD mortality, we aimed to examine the prospective associations between OPA and CVD mortality using a large representative sample of adult workers in the US. Furthermore, evidence showed that low socioeconomic status (SES) was associated with higher risk of CVD [14]. In addition, it has been suggested that SES (e.g., education) could moderate the associations of adverse work environment with health (i.e., stronger effects of poor working conditions on health among people with low SES) [15]. However, only one cohort study examined associations of OPA with CVD stratified by SES in Denmark, which showed that education and income did not modify associations between OPA and CVD [5]. Thus, we also aimed to examine whether the associations of OPA with CVD mortality were different by SES in the US.

## Methods

### Study design and population

Participants aged ≥ 18 years (*n* = 44,233) were selected from the National Health Interview Survey (NHIS) 1988, an annual household interview survey representative of the non-institutionalized civilian population across the US. NHIS used multistage probability sampling procedure to select participants and conducted the survey since 1957 [16]. We excluded participants who did not work or who were unknown if working in past year (*n* = 14,143), had missing data on strenuous OPA (e.g., lifting, pushing, or pulling heavy objects) (*n* = 1,187), were ineligible for mortality data (*n* = 297), or had inaccurate follow up length (*n* = 2). Finally, 28,604 participants were included in this analysis (Supplementary Fig. 1). National Center for Health Statistics Ethics Review Board approved NHIS study protocol. As NHIS is de-identified, publicly available data, this study was exempt from University of California, Los Angeles (UCLA) Institutional Review Board review. Informed consent was obtained from all subjects involved in the study. The manuscript was written according to the Strengthening the Reporting of Observational Studies in Epidemiology (STROBE) guidelines.

### Assessment of occupational physical activity

OPA was assessed at baseline via the self-administrated questionnaire [17]. Participants were asked on both intensity and duration “Did your job require you to do repeated strenuous physical activities such as lifting, pushing or pulling heavy objects?” and “How many minutes per week did you typically spend doing strenuous physical exercise at work?”. Participants then reported their average weekly time (in minutes) spent on strenuous OPA (e.g., lifting, pushing, or pulling heavy objects). For the primary analysis, we treated the strenuous OPA as a categorical variable, which was none (0 min/week), low (> 0 and < 50 min/week, reference group), medium (≥ 50 and < 150 min/week), and high (≥ 150 min/week), given the facts, (i) 69.1% of strenuous OPA were 0, then we created a separate category for none strenuous OPA; (ii) we chose the OPA group which had the lowest CVD mortality rate as the reference group (i.e., 0 < OPA < 50 min/week); (iii) CVD mortality rate increased rapidly when OPA ≥ 150 min/week (Supplementary Fig. 2).

### Assessment of mortality

We ascertained cases of death by the International Classification of Diseases, Tenth Version (ICD–10) with a linkage of NHIS data to the National Death Index (NDI) through 31 December 2019 [18]. For this study, the primary outcome was total CVD mortality (i.e., classified using ICD–10 codes: I00 to I09, I11, I13, I20 to I51, and I60 to I69). In sensitivity analysis, we also assessed all-cause mortality and separated heart disease mortality (i.e., classified using ICD-10 codes: I00 to I09, I11, I13, and I20 to I51).

### Covariates

Sociodemographic information, including age (years), sex (male or female), race/ethnicity (White or non-White), marital status (married/living with a partner, widowed/divorced/separated, or never married), education (high school or less, or higher than high school degree), annual household income (<$30,000, or ≥$30,000), and occupation type (manual or non-manual workers), were self-reported from questionnaires [16, 19]. Health status (i.e., pre-existing cardiometabolic disorders, yes or no), lifestyles [i.e., smoking (never, former, current), alcohol drinking (never, former, current)], and weight (kg) and height (m) were also assessed from interview questionnaires [16, 19]. Body mass index (BMI) was calculated using weight and height (weight in kg/[height in m]^2^). Cardiometabolic disorders were classified based on self-reported diagnoses. Specifically, participants were classified as having pre-existing cardiometabolic disorders if they reported having either heart disease, hypertension, diabetes, or obesity. We did not use dyslipidemia [20] to define cardiometabolic disorders as it was not assessed in the survey.

### Statistical analysis

Due to the sampling procedure and survey design, we applied sampling weights to all analyses according to the NHIS analytical guide [16], so the results could be generalizable to the US workers. We conducted analyses using SAS version 9.4 (SAS Institute, Cary, NC, USA) and R version 4.2.1 and considered the statistically significant level as a two-sided $\:{\upalpha\:}$ level < 0.05.

We presented baseline characteristics of participants, reported the distribution of strenuous OPA and CVD mortality rate. For descriptive analyses, we reported the weighted percentage and actual frequency [% (N)] for categorical variables and weighted mean and standard errors (SE) for continuous variables. We used the chi-square test for categorical variables and one-way analysis of variance for continuous variables to compare between OPA groups.

We examined the association of OPA with total CVD mortality using Cox proportional hazards regression models. The proportional hazards assumption was verified using Schoenfeld residuals. Following unadjusted models, adjusted models controlled for confounders of age, sex, race/ethnicity, marital status, education, annual household income, occupation type, pre-existing cardiometabolic disorders (Supplementary Fig. 3) [10, 21, 22]. Since each adjusted covariate had less than 11% missing data, we conducted the primary analysis using complete case data, including only participants with non-missing values for all adjusted covariates.

In sensitivity analysis, we first examined associations of OPA with all-cause mortality and heart disease mortality. Second, we categorized strenuous OPA into four levels: none, low (> 0 and < 112 min/week), medium (≥ 112 and < 330 min/week), and high (≥ 330 min/week). These categories were based on the tertile distribution of non-zero OPA time. Then we examined associations of tertile distribution classified OPA with total CVD mortality adjusting for same confounders. Third, we examined the associations of OPA with total CVD mortality excluding deaths within 5 years. Fourth, we treated the missing value as a separate category in adjusted models. Fifth, we further controlled for potential mediators of smoking, alcohol drinking, and BMI (Supplementary Fig. 3).

Finally, we examined the associations of OPA with total CVD mortality stratified by education and annual household income. In the sensitivity analysis, we also examined the associations of OPA with CVD mortality stratified by pre-existing cardiometabolic disorders. We calculated P values for the interaction effect of OPA and education, OPA and income, OPA and pre-existing cardiometabolic disorders on total CVD mortality using the likelihood ratio test.

## Results

### Characteristics of study participants

Among 28,604 participants, 69.1% (*n* = 20150), 3.4% (*n* = 988), 8.2% (*n* = 2,293), and 19.3% (*n* = 5,173) had none, low, medium and high strenuous OPA. Workers with high strenuous OPA were the youngest (35.38 years), had the highest percentages of high school or less degree (73.6%) and annual household income < $30,000 (59.8%), as well as the lowest percentages of females (28.7%) and non-manual workers (38.3%) (Table 1).

**Table 1:** Characteristics of participants by strenuous occupational physical activity (OPA) in the National Health Interview Survey (NHIS) 1988

Characteristics	Strenuous OPA									Overall (*N* = 28604)	
	None [69.1% (20150)]		Low [3.4% (988)]		Medium [8.2% (2293)]		High [19.3% (5173)]		*P*-values		
Age (years)	38.78	(0.14)	36.01	(0.51)	36.79	(0.34)	35.38	(0.21)	< 0.001	37.86	(0.13)
Female, % (N)	51.9	(11442)	51.1	(557)	36.7	(946)	28.7	(1745)	< 0.001	46.2	(14690)
White, % (N)	85.8	(16917)	87.5	(849)	90.4	(2024)	85.4	(4327)	< 0.001	86.2	(24117)
High school or less, % (N)	47.7	(9373)	52.8	(509)	63.9	(1403)	73.6	(3736)	< 0.001	54.2	(15021)
Marital Status, % (N)									< 0.001		
Married/living with partner	66.3	(11419)	59.9	(519)	65.8	(1312)	64.4	(2951)		65.7	(16201)
Widowed/Divorced/Separated	12.9	(4050)	12.1	(181)	11.4	(421)	11.9	(950)		12.6	(5602)
Never married	20.7	(4665)	27.9	(288)	22.8	(558)	23.7	(1267)	< 0.001	21.7	(6778)
Annual household income <$30,000, % (N)	42.9	(9141)	50.0	(546)	51.6	(1232)	59.8	(3069)		47.1	(13988)
Non-manual workers, % (N)	77.4	(15889)	71.4	(726)	56.8	(1336)	38.3	(2138)	< 0.001	68.0	(20089)
Pre-existing cardiometabolic disorders, % (N)	25.4	(5075)	27.2	(259)	26.8	(629)	27.1	(1403)	0.07	25.9	(7366)
Alcohol drinking, % (N)									0.04		
Never	14.5	(2854)	14.1	(134)	12.1	(264)	12.9	(638)		14.0	(3890)
Former	26.4	(5262)	28.5	(285)	26.6	(623)	26.5	(1345)		26.5	(7515)
Current	59.1	(11865)	57.3	(568)	61.2	(1385)	60.6	(3146)		59.5	(16964)
Cigarettes smoking, % (N)									< 0.001		
Never	50.6	(10041)	51.7	(497)	43.6	(977)	40.2	(2011)		48.1	(13526)
Former	22.8	(4475)	23.1	(219)	21.4	(477)	20.3	(1021)		22.2	(6192)
Current	26.6	(5481)	25.2	(265)	35.0	(816)	39.5	(2090)		29.7	(8652)
Body mass index (kg/m^2^)	24.66	(0.04)	24.63	(0.15)	25.11	(0.09)	25.22	(0.07)	< 0.001	24.80	(0.33)

Data were presented as weighted percentage, % (actual frequency, N) and weighted mean (standard error, SE) for categorical and continuous variables, respectively

The strenuous OPA was categorized as none, low (> 0 and < 50 min/week), medium (≥ 50 and < 150 min/week], and high (≥ 150 min/week)]

P-values were compared between four strenuous OPA groups using χ2-tests and one-way ANOVA for categorical and continuous variables, respectively

Participants were classified as having pre-existing cardiometabolic disorders if they reported having either heart disease, hypertension, diabetes, or obesity. We did not use dyslipidemia to define cardiometabolic disorders as it was not assessed in the survey

NHIS, National Health Interview Survey; OPA, occupational physical activity

### Associations of OPA with CVD mortality

During a median follow-up time of 31.24 years, 2213 participants died from CVD (273.30 per 100,000 person-years). The unadjusted CVD mortality rates in participants with none, low, medium and high strenuous OPA were 277.39, 188.89, 250.44, and 283.94 per 100,000 person-years, respectively (Table 2). In unadjusted models, compared to workers with low OPA, workers with none, medium and high OPA had higher risk of total CVD mortality, which was a U-shape. After adjusting for confounders of age, sex, race/ethnicity, education, marital status, annual household income, occupation type, and pre-existing cardiometabolic disorders, we still found similar U-shapes for total CVD mortality. The adjusted hazard ratios (HRs), 95% confidence intervals (CIs) and P-values of the none, low, medium, and high OPA groups were 1.39 (1.01–1.91, *P* = 0.04), 1.00 (reference), 1.18 (0.83–1.66, *P* = 0.36) and 1.58 (1.12–2.22, *P* = 0.01) for CVD mortality. In sensitivity analyses, we also found similar U-shaped associations of OPA with all-cause and heart disease mortality (Supplementary Table 1). The pattern of associations of OPA with CVD mortality remained stable although some estimates were statistically insignificant after using tertile distribution based OPA as the exposure (Supplementary Table 2), or deleting death within 5 years (Supplementary Table 3), or creating a separate category for missing covariates (Supplementary Table 4). After adjusting for potential mediators, the elevated CVD mortality in the high OPA group was reduced, and the increased mortality in the none OPA group also decreased and was no longer statistically significant (Supplementary Table 5).

**Table 2:** Associations of occupational strenuous physical activities with total cardiovascular disease (CVD) mortality in the National Health Interview Survey (NHIS) 1988 till 2019

			CVD mortality							
	Cases/person years	Crude mortality rate (per 100,000 person years)	Unadjusted model (*n* = 28604)				Adjusted model (*n* = 24895)			
			HR	95%CI		*P*	HR	95%CI		*P*
Occupational strenuous physical activities										
None	1581 / 569948.25	277.39	**1.50**	**(1.12**,	**2.00)**	**0.01**	**1.39**	**(1.01**,	**1.91)**	**0.04**
Low (> 0 and < 50 min/week)	54 / 28587.75	188.89	1.00				1.00			
Medium (≥ 50 and < 150 min/week)	162 / 64685.42	250.44	1.25	(0.91,	1.71)	0.16	1.18	(0.83,	1.66)	0.36
High (≥ 150 min/week)	416 / 146512.33	283.94	**1.53**	**(1.12**,	**2.09)**	**0.01**	**1.58**	**(1.12**,	**2.22)**	**0.01**

Cox proportional hazard regression model was used. Hazard ratio, 95% CI, and P-values were reported. Boldface indicated statistical significance (*P* < 0.05)

Sampling weights were applied

The strenuous OPA was categorized as none, low (> 0 and < 50 min/week, reference group), medium (≥ 50 and < 150 min/week), and high (≥ 150 min/week)

CVD mortality included heart disease and cerebrovascular diseases mortality

Unadjusted model: Crude model. Adjusted model: Adjusted for age, sex, race/ethnicity, marital status, education, annual household income, occupation type, and pre-existing cardiometabolic disorders

CVD, cardiovascular disease; NHIS, National Health Interview Survey; OPA, occupational physical activity

**Fig. 1:**
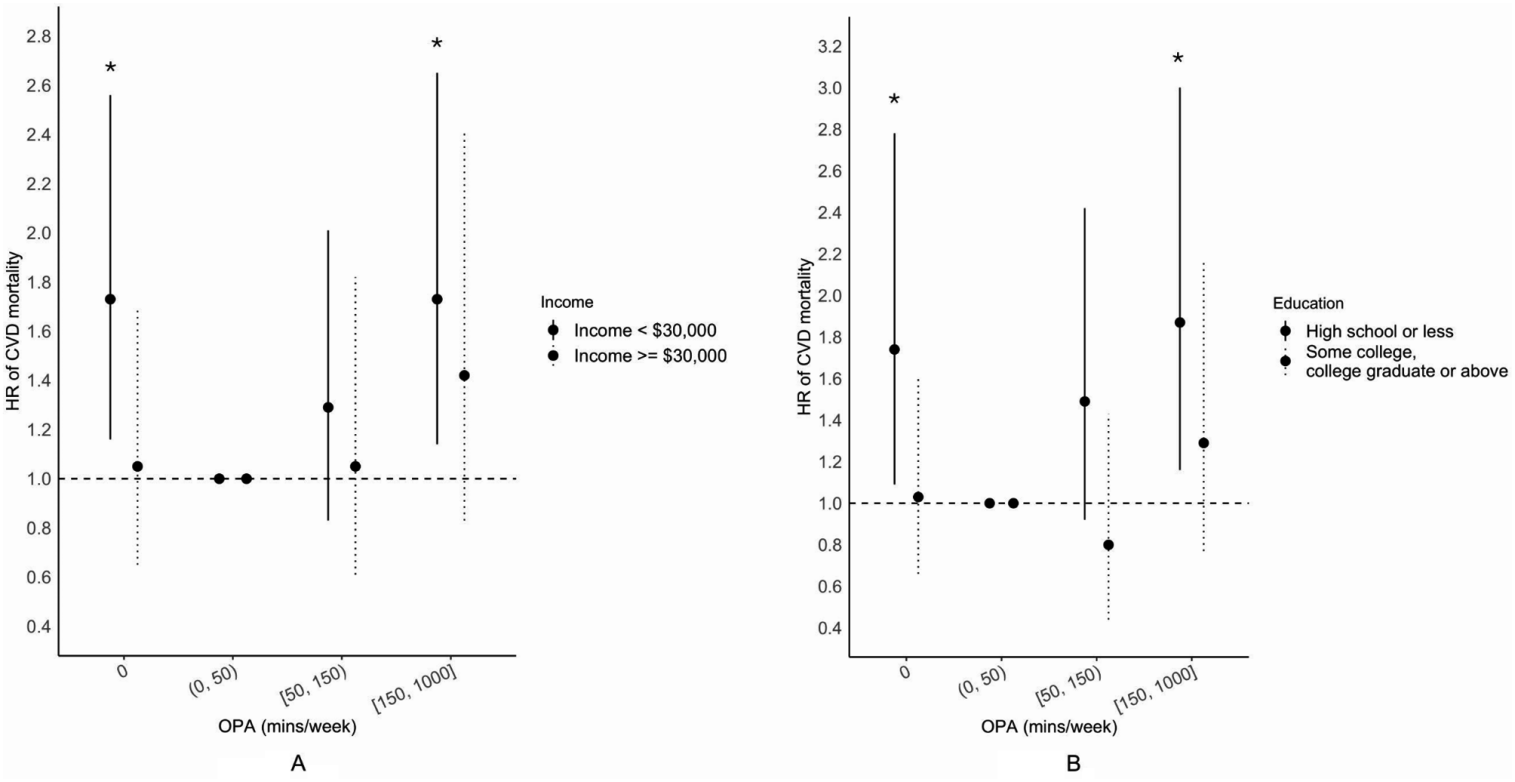
Associations of occupational strenuous physical activities with cardiovascular disease (CVD) mortality stratified by education (**A**) and annual household income (**B**) in the National Health Interview Survey (NHIS) 1988 till 2019. Cox proportional hazard regression model was used. * indicated statistical significance (*P* < 0.05). Sampling weights were applied. The strenuous OPA was categorized as none, low (> 0 and < 50 min/week, reference group), medium (≥ 50 and < 150 min/week), and high (≥ 150 min/week). CVD mortality included heart disease and cerebrovascular diseases mortality. Adjusted for age, sex, race/ethnicity, marital status, education, annual household income, occupation type, and pre-existing cardiometabolic disorders. When stratified by education or annual household income, that variable was not adjusted for. CVD, cardiovascular disease; NHIS, National Health Interview Survey; OPA, occupational physical activity

When stratified by education, the associations between OPA and total CVD mortality were stronger in workers with high school or less education [estimates of the none, low, medium, and high OPA groups were 1.74 (1.09–2.78, *P* = 0.02), 1.00 (reference), 1.49 (0.92–2.42, *P* = 0.11), and 1.87 (1.16–3.00, *P* = 0.01) for workers with high school or less education; and 1.03 (0.66–1.60, *P* = 0.91), 1.00 (reference), 0.80 (0.44–1.43, *P* = 0.44), and 1.29 (0.77–2.16, *P* = 0.34) for workers with higher than high school education]. In addition, the associations between OPA and total CVD mortality were more pronounced in workers with annual household income <$30,000 [estimates of the none, low, medium, and high OPA groups were 1.73 (1.16–2.56, *P* = 0.01), 1.00 (reference), 1.29 (0.83–2.01, *P* = 0.26), and 1.73 (1.14–2.65, *P* = 0.01) for workers having annual household income <$30,000; and 1.05 (0.65–1.70, *P* = 0.84), 1.00 (reference), 1.05 (0.61–1.82, *P* = 0.86), and 1.42 (0.83–2.42, *P* = 0.20) for workers having annual household income ≥$30,000] (Fig. 1). When stratified by pre-existing cardiometabolic disorders, the associations between OPA and total CVD mortality were stronger in workers without cardiometabolic disorders [estimates of the none, low, medium, and high OPA groups were 1.81 (1.06, 3.07, *P* = 0.03), 1.00 (reference), 1.71 (0.96, 3.06, *P* = 0.07), and 2.04 (1.19, 3.51, *P* = 0.01) for workers without cardiometabolic disorders; and 1.17 (0.78, 1.78, *P* = 0.44), 1.00 (reference), 0.91 (0.58, 1.44, *P* = 0.69), and 1.33 (0.85, 2.08, *P* = 0.22) for workers with cardiometabolic disorders] (**Supplementary Table 6**). Tests for interactions were all significant (all *Ps* < 0.001).

## Discussion

In this prospective study with nationally representative sample of US workers, we found U-shaped association between strenuous OPA and CVD mortality, with higher risks at both none and high levels of strenuous OPA, independently from demographic factors, SES, and pre-existing cardiometabolic disorders. Particularly, the associations of strenuous OPA with CVD mortality were stronger in workers with low SES (i.e., lower education or income levels). With nearly 70% of workers reporting no OPA and about 20% engaging in high levels of strenuous OPA, our findings emphasize the need for workplace interventions that target both the lack of OPA and excessive strenuous OPA. Potential strategies could include promoting moderate physical activity [23], adjusting workload intensity and duration, and implementing health monitoring programs to reduce CVD mortality risks among workers.

Up to now, only one study assessed associations between OPA and CVD mortality in the US [13]. The study found the J-shaped association of years of heavy labor OPA with CVD mortality [HR = 1 (reference), 0.94 (0.87–1.03), 0.89 (0.83–0.95), 0.98 (0.93–1.04), 1.03 (0.96–1.10), 1.10 (1.06–1.15), 1.02 (0.98–1.05) in individuals with 0, < 1, 1–2, 3–5, 6–9, $\:\ge\:$ 10 years of heavy labor OPA, respectively] among retired males aged 50–71 years in the US [13], which was similar to our study, however, this study only included retirees, introducing potential selection bias by excluding individuals who did not reach retirement, potentially underrepresenting severe occupational exposures or early health outcomes. Our study was the first to examine the prospective association between baseline time spent on OPA and CVD mortality in a large nationally representative sample of workers ≥ 18 years in the US. Findings from our study also aligned with a longitudinal study in Norway among 17,697 participants aged 20–62 years which found a U-shaped association between OPA and CVD mortality [24]. The different findings compared to previous studies which found inverse [9, 10] or null [11, 12] associations of OPA with CVD mortality may be attributed to discrepant measurements of OPA. We compared different levels of time on strenuous OPA, while previous studies compared OPA by levels of walking, lifting, and heavy labor work (e.g., sedentary work, work with some walking, work with walking and lifting, or work with heavy manual lab) [9–11] or by frequency (e.g., from never to very often) on OPA [12]. Future studies should aim to use standardized and consistent methods for measuring OPA, such as uniform categories for activity types or standardized metrics for activity duration and frequency, to facilitate comparison across studies.

The possible explanations for the U-shaped associations in our study may be due to two facets. Compared to workers with low strenuous OPA, workers without strenuous OPA had more sedentary lifestyles, which increased BMI, inflammation, blood pressure and triglycerides, then increased CVD and mortality [25–28]. On the other hand, high levels on strenuous OPA with long duration, heavy lifting, pushing, pulling and static postures, and insufficient recovery, led to increased heart rate, inflammation, and blood pressure, finally increasing CVD mortality [29–31]. In addition, workers with high strenuous OPA may have less LTPA and more alcohol drinking and smoking, resulting in increased CVD mortality [4, 32]. Some explanations are supported by the attenuated associations between OPA and CVD mortality after additional adjustment for mediators such as alcohol consumption, smoking, and BMI (Supplementary Table 5). The similar associations across CVD, heart disease, and all-cause mortality highlight the potential shared underlying mechanisms, such as cardiovascular strain and systemic inflammation, which may contribute to mortality risk at the extremes of strenuous OPA. This consistency reinforces the importance of balanced OPA for optimal health.

We only found one cohort study in Denmark examining associations of OPA with CVD stratified by SES [5]. Using the Copenhagen General Population Study data among 104,046 participants aged 20–100 years, it found that education and income did not modify associations between OPA and CVD [5]. The discrepancies between their findings and ours may be due to variations in OPA measurement: the Copenhagen study assessed OPA by intensity levels (e.g., walking, lifting, heavy labor), while our study focused on the duration of strenuous OPA. Further studies using standardized OPA assessment methods are needed to improve comparability across studies. In addition, macro-level labor and social policies may also help explain the difference. Denmark’s stronger performance on six indicator, such as higher active labor market policy expenditures, investments in rehabilitation services, and lower income inequality [33], suggests a more protective environment for workers, particularly those with low SES. These indicators represent resources that likely mitigate the adverse effects of strenuous work conditions on health. For example, higher union density and robust support for unemployed individuals in Denmark may reduce stress and promote job security, thereby lessening the health risks associated with physically demanding jobs. This protective social environment could attenuate the association between OPA and CVD in Denmark, especially among lower SES groups, compared to the US setting. Nevertheless, our results stratified by education and income were consistent with previous studies assessing associations of other work-related factors, such as working hours, with CVD and CVD mortality moderated by SES [34, 35]. For example, a meta-analysis of cohort studies among 768,751 participants found positive associations of working hours (working ≥ 55 h/week vs. working 35–40 h/week) with heart disease mortality in workers with low SES [HR = 1.43 (1.14–1.79)] but not in workers with intermediate [HR = 1.10 (0.78–1.55)] or high SES [HR = 0.94 (0.72–1.21)] [34]. The mechanisms of the stronger associations between OPA and CVD mortality in low education or income groups may be because that workers with low education or low income are more likely to engage in unhealthy behaviors [36, 37], experience higher-intensity OPA, and work in more hazardous environments with greater stress. They also tend to have lower levels of decision-making authority, fewer resources to manage work-related stress, and limited access to healthcare, treatment options, and social support [36, 38–40]. Interestingly, the effort-reward imbalance theory suggests that a mismatch between high job demands and low rewards (e.g., low income) can elevate CVD risk due to chronic stress. This aligns with our findings, where the OPA-CVD mortality association was stronger in lower-income workers, who may face greater stress from high physical demands and limited rewards, contributing to their increased cardiovascular risk [41–43].

We found the association between OPA and CVD mortality was stronger in workers without pre-existing cardiometabolic disorders. This may be because individuals with baseline cardiometabolic disease were more likely to receive routine medical interventions that could obviously reduce the risk of CVD mortality even they were engaged in strenuous OPA. In contrast, those without baseline disease may lack these protective factors, leaving them more susceptible to the potential harms of excessive OPA. However, it is a particular limitation of our study that we did not measure medication use. We only found one cohort study in Denmark examining associations of OPA with CVD stratified by baseline cardiometabolic disorders [5]. It reported similar associations in those with or without diabetes, and with high or low levels of blood pressure and lipids. The differences between our findings and theirs may stem from different OPA measures and Copenhagen study controlled for blood pressure medication.

### Strengths and limitations

Our study had lots of strengths. We investigated the association between OPA and CVD mortality in a nationally representative sample of workers. Notably, the study had large sample size. Additionally, both exposure misclassification and selection bias are common challenges in this type of OPA research [21, 22] and we tried to reduce these issues. Compared to the previous study in the US focusing solely on retirees, we included participants aged ≥ 18 years, thereby minimizing selection bias. Meanwhile, the NHIS OPA data provided information on both intensity and duration, enabling a more accurate operationalization of OPA exposure compared to previous studies only assessing OPA by intensity levels, potentially reducing exposure misclassification. Furthermore, Our findings are robust, as shown by consistent association patterns between OPA and CVD mortality across sensitivity analyses, such as using tertile-based OPA, excluding early deaths or categorizing missing covariates separately. Nonetheless, the study also had limitations. First, we may have residual confounding like other observational studies. For example, some environmental factors (e.g., chemicals, sunlight exposure, dust, and working hours etc.) and psychological factors were not measured. If individuals in high OPA were exposed to harmful environmental factors, this could exaggerate the observed association between OPA and CVD mortality. Without adjusting for these factors, our findings might overestimate the effect of OPA on CVD outcomes, as some of the risk attributed to OPA could actually be due to these unmeasured environmental exposures. Second, OPA was only assessed at the strenuous level, no light or moderate OPA was measured, leaving the largest group of none-OPA in this study (69.1%); furthermore, OPA in the NHIS was assessed at baseline only. Thus, the inaccurate measurement of exposure would not be able to rule out when considering the different levels of OPA, and changes in OPA over time. For instance, pre-existing cardiometabolic disorders might drive workers to exit from labor market earlier, discontinuing exposure to strenuous OPA. Thus, multi-wave data collection with repeated measures of employment status and OPA would be optimal to improve research quality for future studies. Third, OPA measure may be influenced by physical capacity. Fitter individuals may engage more in strenuous OPA, while less fit individuals may underreport or avoid it. This could result in an overestimation of the association between OPA and CVD mortality in fitter individuals and an underestimation in less fit populations, limiting the generalizability of our findings across different fitness levels.

## Conclusion

In this large prospective cohort study with nationally representative workers, we found U-shaped associations of strenuous OPA with CVD mortality; importantly, the associations were more pronounced in workers with low SES. Our findings have practical implications for the US workforce engaged in none and high strenuous OPA, suggesting a need for workplace policies to balance physical workload and support cardiovascular health monitoring. Further research is essential, both in the US and globally, especially in non-Western countries with a higher prevalence of strenuous OPA. Expanding this research will enhance the relevance of our findings and support culturally tailored occupational health interventions.

## Electronic supplementary material

Below is the link to the electronic supplementary material.

Supplementary Material 1

## Data Availability

Publicly available datasets were analyzed in this study. This data can be found here: https://www.cdc.gov/nchs/nhis/index.htm.

## Abbreviations

AARP: American Association of Retired Persons
CVD: cardiovascular disease
LTPA: leisure time physical activity
NDI: National Death Index
NHIS: National Health Interview Survey
OPA: occupational physical activity
SE: standard error
SES: socioeconomic status
STROBE: Strengthening the Reporting of Observational Studies in Epidemiology
UCLA: University of California, Los Angeles
US: United States
