# Supplementary material for: Occupational physical activity and cardiovascular disease mortality in the United States, 1988–2019
Source: BMC Public Health. 2025 Jan 7;25:77. doi: 10.1186/s12889-024-21225-x (PMC11708131; doi:10.1186/s12889-024-21225-x)
Supplement: Supplementary file 1 — Supplementary Material 1 [file 12889_2024_21225_MOESM1_ESM.docx]

**Supplementary material**

**Occupational physical activity and cardiovascular disease mortality in the United States, 1988-2019**

**Supplementary Fig. 1**. Participants flow chart

NHIS, National Health Interview Survey

**Supplementary Fig. 2**. Plot of crude cardiovascular disease (CVD) mortality rate with occupational strenuous physical activities

CVD, cardiovascular disease; OPA, occupational physical activity

**Supplementary Fig. 3.** Directed acyclic graph of strenuous occupational physical activity (OPA) with cardiovascular disease (CVD) mortality

The X refers to exposure. The Y refers to outcome. The C refers to confounders. The M refers to mediators. The black texts were measured variables. The gray texts were unmeasured variables.

CVD, cardiovascular disease; LTPA, leisure-time physical activity; OPA, occupational physical activity; SES, socioeconomic status

**Supplementary Table 1**. Associations of occupational strenuous physical activities with all-cause mortality and separated

heart disease mortality in National Health Interview Survey (NHIS) 1988 till 2019

|  |  | **All-cause mortality** | | | | |
| --- | --- | --- | --- | --- | --- | --- |
|  | **Cases/person years** | **Crude mortality rate (per 100,000 person years)** | **HR** | **95%CI** | | ***P*** |
| Occupational strenuous physical activities |  |  |  |  |  |  |
| None | 5340 / 569948.25 | 936.93 | 1.17 | (0.97, | 1.40) | 0.10 |
| Low (>0 and < 50 minutes/week) | 219 / 28587.75 | 766.06 | 1.00 |  |  |  |
| Medium (≥ 50 and < 150 minutes/week) | 625 / 64685.42 | 966.22 | 1.17 | (0.95, | 1.46) | 0.15 |
| High (≥ 150 minutes/week) | 1386 / 146512.33 | 946.00 | **1.27** | **(1.05,** | **1.53)** | **0.01** |
|  |  | **Heart disease mortality** | | | | |
|  |  | **Crude mortality rate (per 100,000 person years)** | **HR** | **95%CI** | | ***P*** |
| Occupational strenuous physical activities |  |  |  |  |  |  |
| None | 1319 / 569948.25 | 231.43 | **1.53** | **(1.04,** | **2.25)** | **0.03** |
| Low (>0 and < 50 minutes/week) | 40 / 28587.75 | 139.92 | 1.00 |  |  |  |
| Medium (≥ 50 and < 150 minutes/week) | 142 / 64685.42 | 219.52 | 1.32 | (0.90, | 1.94) | 0.16 |
| High (≥ 150 minutes/week) | 347 / 146512.33 | 236.84 | **1.74** | **(1.16,** | **2.62)** | **0.01** |
| Cox proportional hazard regression model was used. Hazard ratio, 95% CI, and P-values were reported. Boldface indicated statistical significance (P<0.05).  Sampling weights were applied.  The strenuous OPA was categorized as none, low (>0 and <50 minutes/week, reference group), medium (**≥**50 and <150 minutes/week), and high (**≥**150 minutes/week).  Adjusted for age, sex, race/ethnicity, marital status, education, annual household income, occupation type, and pre-existing cardiometabolic disorders.  NHIS, National Health Interview Survey; OPA, occupational physical activity | | | | | | |

**Supplementary Table 2**. Associations of tertile distribution based occupational strenuous physical activities with

total cardiovascular disease (CVD) mortality in National Health Interview Survey (NHIS) 1988 till 2019

|  | **CVD mortality (n=24895)** | | | |
| --- | --- | --- | --- | --- |
|  | **HR** | **95%CI** | | ***P*** |
| Occupational strenuous physical activities |  |  |  |  |
| None | 1.17 | (0.93, | 1.47) | 0.19 |
| Low (>0 and < 112 minutes/week) | 1.00 |  |  |  |
| Medium (≥ 112 and < 330 minutes/week) | 1.08 | (0.81, | 1.44) | 0.59 |
| High (≥ 330 minutes/week) | **1.38** | **(1.05,** | **1.82)** | **0.02** |
| Cox proportional hazard regression model was used. Hazard ratio, 95% CI, and P-values were reported. Boldface indicated statistical significance (P<0.05).  Sampling weights were applied.  **The strenuous OPA was categorized as none, low (>0 and <112 minutes/week), medium (≥112 and <330 minutes/week), and high (≥330 minutes/week) based on the tertile distribution of non-zero time of OPA**.  CVD mortality included heart disease and cerebrovascular diseases mortality.  Adjusted for age, sex, race/ethnicity, marital status, education, annual household income, occupation type, and pre-existing cardiometabolic disorders.  CVD, cardiovascular disease; NHIS, National Health Interview Survey; OPA, occupational physical activity | | | | |

**Supplementary Table 3**. Associations of occupational strenuous physical activities with total cardiovascular disease (CVD) mortality in National Health Interview Survey (NHIS) 1988 till 2019 1988 till 2019 excluding deaths within 5 years

|  | **CVD mortality** | | | | | | | | | | | | | | | | | | | |
| --- | --- | --- | --- | --- | --- | --- | --- | --- | --- | --- | --- | --- | --- | --- | --- | --- | --- | --- | --- | --- |
|  | **Exclude death within 1 year (n=28530)** | | | | **Exclude death within 2 years (n=28450)** | | | | **Exclude death within 3 years (n=28356)** | | | | **Exclude death within 4 years (n=28268)** | | | | **Exclude death within 5 years (n=28137)** | | | |
|  | **HR** | **95%CI** | | ***P*** | **HR** | **95%CI** | | ***P*** | **HR** | **95%CI** | | ***P*** | **HR** | **95%CI** | | ***P*** | **HR** | **95%CI** | | ***P*** |
| Occupational strenuous physical activities |  |  |  |  |  |  |  |  |  |  |  |  |  |  |  |  |  |  |  |  |
| None | 1.37 | (1.00, | 1.88) | 0.05 | 1.36 | (0.99, | 1.87) | 0.06 | **1.40** | **(1.01,** | **1.93)** | **0.04** | 1.37 | (1.00, | 1.89) | 0.05 | **1.43** | **(1.03,** | **1.99)** | **0.03** |
| Low (>0 and < 50 minutes/week) | 1.00 |  |  |  | 1.00 |  |  |  | 1.00 |  |  |  | 1.00 |  |  |  | 1.00 |  |  |  |
| Medium (≥ 50 and < 150 minutes/week) | 1.17 | (0.83, | 1.65) | 0.38 | 1.16 | (0.82, | 1.65) | 0.40 | 1.20 | (0.85, | 1.71) | 0.30 | 1.16 | (0.81, | 1.67) | 0.40 | 1.24 | (0.86, | 1.78) | 0.25 |
| High (≥ 150 minutes/week) | **1.58** | **(1.12,** | **2.23)** | **0.01** | **1.56** | **(1.10,** | **2.20)** | **0.01** | **1.60** | **(1.13,** | **2.27)** | **0.01** | **1.59** | **(1.12,** | **2.25)** | **0.01** | **1.66** | **(1.16,** | **2.40)** | **0.01** |
| Cox proportional hazard regression model was used. Hazard ratio, 95% CI, and P-values were reported. Boldface indicated statistical significance (P<0.05).  Sampling weights were applied.  **Deaths within 5 years were excluded.**  The strenuous OPA was categorized as none, low (>0 and <50 minutes/week, reference group), medium (≥50 and <150 minutes/week), and high (≥150 minutes/week).  CVD mortality included heart disease and cerebrovascular diseases mortality.  Adjusted for age, sex, race/ethnicity, marital status, education, annual household income, occupation type, and pre-existing cardiometabolic disorders.  CVD, cardiovascular disease; NHIS, National Health Interview Survey; OPA, occupational physical activity | | | | | | | | | | | | | | | | | | | | |

**Supplementary Table 4**. Associations of occupational strenuous physical activities with total cardiovascular disease (CVD)

mortality in National Health Interview Survey (NHIS) 1988 till 2019 addressing missing of covariates

|  | **CVD mortality (n=28464)** | | | |
| --- | --- | --- | --- | --- |
|  | **HR** | **95%CI** | | ***P*** |
| Occupational strenuous physical activities |  |  |  |  |
| None | 1.31 | (0.96, | 1.78) | 0.09 |
| Low (>0 and < 50 minutes/week) | 1.00 |  |  |  |
| Medium (≥ 50 and < 150 minutes/week) | 1.10 | (0.78, | 1.54) | 0.58 |
| High (≥ 150 minutes/week) | **1.49** | **(1.07,** | **2.06)** | **0.02** |
| Cox proportional hazard regression model was used. Hazard ratio, 95% CI, and P-values were reported. Boldface indicated statistical significance (P<0.05).  Sampling weights were applied.  **As the percentage of annual household income and pre-existing cardiometabolic disorders missing were 10.8% and 3.0%, respectively, a separated category for income and pre-existing cardiometabolic disorders missing was created. Percentages of other covariates missing were all <1%.**  The strenuous OPA was categorized as none, low (>0 and <50 minutes/week, reference group), medium (≥50 and <150 minutes/week), and high (≥150 minutes/week).  CVD mortality included heart disease and cerebrovascular diseases mortality.  Adjusted for age, sex, race/ethnicity, marital status, education, annual household income, occupation type, and pre-existing cardiometabolic disorders.  CVD, cardiovascular disease; NHIS, National Health Interview Survey; OPA, occupational physical activity | | | | |

**Supplementary Table 5**. Associations of occupational strenuous physical activities with total cardiovascular disease (CVD)

mortality in National Health Interview Survey (NHIS) 1988 till 2019 adjusting for potential mediators

|  | **CVD mortality (n=28464)** | | | |
| --- | --- | --- | --- | --- |
|  | **HR** | **95%CI** | | ***P*** |
| Occupational strenuous physical activities |  |  |  |  |
| None | 1.35 | (0.97, | 1.90) | 0.08 |
| Low (>0 and < 50 minutes/week) | 1.00 |  |  |  |
| Medium (≥ 50 and < 150 minutes/week) | 1.14 | (0.79, | 1.64) | 0.48 |
| High (≥ 150 minutes/week) | **1.48** | **(1.03,** | **2.15)** | **0.04** |
| Cox proportional hazard regression model was used. Hazard ratio, 95% CI, and P-values were reported. Boldface indicated statistical significance (P<0.05).  Sampling weights were applied.  The strenuous OPA was categorized as none, low (>0 and <50 minutes/week, reference group), medium (≥50 and <150 minutes/week), and high (≥150 minutes/week).  CVD mortality included heart disease and cerebrovascular diseases mortality.  Adjusted for age, sex, race/ethnicity, marital status, education, annual household income, occupation type, and pre-existing cardiometabolic disorders, **smoking, alcohol drinking and body mass index.**  CVD, cardiovascular disease; NHIS, National Health Interview Survey; OPA, occupational physical activity | | | | |

**Supplementary Table 6**. Associations of occupational strenuous physical activities with total cardiovascular disease (CVD)

mortality in National Health Interview Survey (NHIS) 1988 till 2019, stratified by pre-existing cardiometabolic disorders

|  | **CVD mortality** | | | | | | | | | | | |
| --- | --- | --- | --- | --- | --- | --- | --- | --- | --- | --- | --- | --- |
|  | **Workers without pre-existing cardiometabolic disorders** | | | | | | **Workers with pre-existing cardiometabolic disorders** | | | | | |
|  | **HR** | **95%CI** | | | ***P*** | | **HR** | | **95%CI** | | | ***P*** |
| Occupational strenuous physical activities |  |  | |  | |  | |  | |  |  |  |
| None | **1.81** | **(1.06,** | **3.07)** | | **0.03** | | 1.17 | | (0.78, | | 1.78) | 0.44 |
| Low (>0 and < 50 minutes/week) | 1.00 |  |  | |  | | 1.00 | |  | |  |  |
| Medium (≥ 50 and < 150 minutes/week) | 1.71 | (0.96, | 3.06) | | 0.07 | | 0.91 | | (0.58, | | 1.44) | 0.69 |
| High (≥ 150 minutes/week) | **2.04** | **(1.19,** | **3.51)** | | **0.01** | | 1.33 | | (0.85, | | 2.08) | 0.22 |
| Cox proportional hazard regression model was used. Hazard ratio, 95% CI, and P-values were reported. Boldface indicated statistical significance (P<0.05).  Sampling weights were applied.  The strenuous OPA was categorized as none, low (>0 and <50 minutes/week, reference group), medium (≥50 and <150 minutes/week), and high (≥150 minutes/week).  CVD mortality included heart disease and cerebrovascular diseases mortality.  Adjusted for age, sex, race/ethnicity, marital status, education, annual household income, occupation type**.**  CVD, cardiovascular disease; NHIS, National Health Interview Survey; OPA, occupational physical activity | | | | | | | | | | | | |
